# *Bacillus subtilis* PS-216 Spores Supplemented in Broiler Chicken Drinking Water Reduce *Campylobacter jejuni* Colonization and Increases Weight Gain

**DOI:** 10.3389/fmicb.2022.910616

**Published:** 2022-07-08

**Authors:** Katarina Šimunović, Orhan Sahin, Andi Erega, Polonca Štefanič, Qijing Zhang, Ines Mandic Mulec, Sonja Smole Možina, Anja Klančnik

**Affiliations:** ^1^Department of Food Science and Technology, Biotechnical Faculty, University of Ljubljana, Ljubljana, Slovenia; ^2^Department of Microbiology, Biotechnical Faculty, University of Ljubljana, Ljubljana, Slovenia; ^3^Veterinary Diagnostic and Production Animal Medicine, College of Veterinary Medicine, Iowa State University, Ames, IA, United States; ^4^Veterinary Microbiology and Preventive Medicine, College of Veterinary Medicine, Iowa State University, Ames, IA, United States

**Keywords:** *Campylobacter jejuni*, *Bacillus subtilis*, probiotic, alternative to antibiotics, spore-containing drinking water, broiler chicken

## Abstract

*Campylobacter jejuni* is the leading cause of bacterial gastroenteritis, or campylobacteriosis, in humans worldwide, and poultry serves as a major source of infection. To reduce the risk associated with *C. jejuni* transmission via poultry meat, effective interventions during poultry production are needed, and the use of probiotics is a promising approach. In this study, 15 *Bacillus subtilis* strains were initially screened for their anti-*Campylobacter* activities. *B. subtilis* PS-216 strain demonstrated the best anti-*Campylobacter* activity against 15 *C. jejuni* isolates when examined using *in vitro* co-cultures. To evaluate the suitability of *B. subtilis* PS-216 for probiotic use, its susceptibility to eight clinically important antimicrobials and simulated gastric conditions was investigated. *B. subtilis* PS-216 was sensitive to all of the tested antibiotics. Although vegetative cells were sensitive to gastric conditions, *B. subtilis* PS-216 spores were highly resistant. We further evaluated the use of a *B. subtilis* PS-216 spore preparation (2.5 × 10^6^ CFU/mL water) to prevent and/or reduce *C. jejuni* colonization in broiler chickens *in vivo*. Compared to the untreated group, significantly lower *Campylobacter* counts were detected in caeca of broilers continuously treated with *B. subtilis* PS-216 spores in their drinking water. Furthermore, broilers continuously treated with *B. subtilis* PS-216 spores showed improved weight gain, compared to the control group. Together, these results demonstrate the potential of *B. subtilis* PS-216 for use in poultry to reduce *C. jejuni* colonization and improve weight gain.

## Introduction

Campylobacteriosis is among the most frequently reported bacterial foodborne infection in both the European Union (EU) and the United States, with *Campylobacter jejuni* as the major causative agent. In the last decade, the number of confirmed infections in the EU exceeded 200,000/year. Symptoms of campylobacteriosis include diarrhea, fever and cramps. Death rarely occurs, but complications can increase more than fivefold due to infections with antibiotic-resistant strains (Helms et al., 2005; EFSA and ECDC, 2021a,b).

As *C. jejuni* is a common avian commensal, efforts are being made to tackle the pathogen at its primary source, the reservoir in chickens. Once *Campylobacter* is introduced onto a farm, its fast spread is imminent (Berndtson et al., 1996), and therefore effective control measures are of great importance. These can include pre-harvest measures (e.g., biosecurity, hygiene measures) to prevent *Campylobacter* from entering a farm and to limit its spread, and post-harvest measures (e.g., freezing, hot-water treatment, irradiation, chemical decontamination) to reduce *Campylobacter* after animal slaughter (Sahin et al., 2015). Reduction of *Campylobacter* in the chicken intestine can significantly reduce the public health risk, and thus the control of *Campylobacter* in poultry production is considered one of the most effective strategies for intervention (Meunier et al., 2016; Dogan et al., 2019; Koutsoumanis et al., 2020).

Probiotics can be used as a pre-harvest measure for pathogen control on poultry farms (Sahin et al., 2015; Alagawany et al., 2018; Koutsoumanis et al., 2020). Probiotics can have multiple beneficial effects on poultry, such as growth promotion, immunomodulation and inhibition of pathogens. The modes of action of probiotic bacteria against pathogens can include production of organic acids and antibacterial substances, competitive exclusion of pathogens, modulation of the host immune system, and others (reviewed by El-Hack et al., 2020; Jha et al., 2020). The main bacteria studied and used as probiotics in poultry are *Lactobacillus* spp., *Bifidobacterium* spp., *Bacillus* spp., *Streptococcus* spp., and *Enterococcus* spp. (Hong et al., 2005; Lutful Kabir, 2009; Alagawany et al., 2018).

*Bacillus subtilis* as a poultry probiotic that is included in commercial formulations, such as GalliPro (Chr Hansen), Calsporin (ORFFA), and Alterion (Novozymes). The administration in chickens of various *B. subtilis* strains either alone or in combination with other bacteria can improve feed conversion and body weight, reduce lesions caused by *Clostridium perfringens*, elongate intestinal villi in necrotic enteritis, modulate the microbiota to improve intestinal *Lactobacillus* numbers, and reduce the number of pathogens such as *C. perfringens*, *Escherichia coli*, *Salmonella enteritidis* and others (Fritts et al., 2000; Jayaraman et al., 2013; Zhou et al., 2015; Hmani et al., 2017; Park et al., 2017; Hayashi et al., 2018). Probiotic effects of different *B. subtilis* strains against *C. jejuni* in poultry have been well documented, although their anti-*Campylobacter* effects were shown to be variable and strain specific (Saint-Cyr et al., 2016), which indicates that not every *B. subtilis* treatment will result in reduced *C. jejuni* counts in the chicken intestine. Therefore, effective and reliable *B. subtilis* probiotics that carry anti-*Campylobacter* traits remain to be developed.

The aim of this study was to initially test different *B. subtilis* strains for their *in vitro* anti-*Campylobacter* activities, and then to apply the best one to *in vivo* testing in broiler chickens. From the 15 *B. subtilis* strains tested against *C. jejuni* NCTC 11168 in co-cultures, *B. subtilis* PS-216 showed the best anti-*Campylobacter* activity and was chosen for further evaluation. In addition to examining its anti-*Campylobacter* activity against different *C. jejuni* strains, *B. subtilis* PS-216 was evaluated for its antimicrobial resistance profile and its survival in the gut environment, using simulated intestinal conditions *in vitro*. Finally, we evaluated the use of *B. subtilis* PS-216 spores to prevent or reduce *C. jejuni* colonization in broiler chickens, and as well as their influence on the broiler weight gain. These results show that when given continuously as a water additive in broilers, *B. subtilis* PS-216 spores can reduce *C. jejuni* colonization in the broiler intestine and increased broiler body weight.

## Materials and Methods

### Animal Ethics

All of the animal protocols and procedures used in this study were reviewed and approved by the Institutional Animal Care and Use Committee at Iowa State University before the start of the experiments (IACUC Protocol IACUC-18-322).

### Strains and Growth Conditions

*Campylobacter jejuni* isolates were derived from human feces, surface water and slaughter house environments, and were previously characterized by Kovač et al. (2018). *B. subtilis* strains were isolated from riverbank soil from Sava River in Slovenia, and were characterized by Štefanič and Mandic Mulec (2009). *B. subtilis* strains from tomato rhizosphere were characterized by Oslizlo et al. (2015) (Supplementary Table 1).

The bacterial strains were stored in 20% glycerol (Kemika, Croatia) and 80% Mueller Hinton (MH) broth (Oxoid, United Kingdom) at –80°C. *C. jejuni* strains were revitalized on Karmali agar (Oxoid, United Kingdom) supplemented with Karmali selective supplement (SR0167E; Oxoid, United Kingdom), with incubation at 42°C in a anaerobic jar flushed with tri-gas mixture (5% O_2_, 10% CO_2_, 85% N_2_) to provide microaerobic conditions for 24 h. *B. subtilis* strains were revitalized on MH agar (BD Difco, United States), with incubation at 37°C under aerobic conditions. The second passage was prepared as an overnight culture (16 h incubation) and was used in the experiments, as described below. To enumerate *C. jejuni* in monocultures and co-cultures, Karmali agar (Oxoid, United Kingdom) supplemented with Karmali selective supplement (SR0167E; Oxoid, United Kingdom) was used. For enumeration of *C. jejuni* from fecal samples, MH agar was supplemented with Bolton broth selective supplement (SR0183; Oxoid, United Kingdom) and growth supplement (SR0232E; Oxoid, United Kingdom) (MH-BSS). For enumeration of *Bacillus* sp. from fecal samples, HiChrom *Bacillus* agar (Himedia, United States) was used.

### Co-cultivation of *Bacillus subtilis* With *Campylobacter jejuni* in Mueller Hinton Broth

Fifteen different *Bacillus subtilis* strains (Supplementary Table 1) were co-cultivated in MH broth with *C. jejuni* 11168 at a 1:10 starting ratio (5 × 10^4^ CFU/mL: 5 × 10^5^ CFU/mL) in favor of *C. jejuni*. Co-cultures and control monocultures were cultivated at 42°C under microaerobic conditions for 24 h. *B. subtilis* strain PS-216 was chosen for the further experiments on the basis of showing the greatest anti-*Campylobacter* effects, as shown by the highest *C. jejuni* reduction in co-culture.

The anti-*Campylobacter* effects of *B. subtilis* PS-216 were further tested on 15 different *C. jejuni* strains isolated from a slaughterhouse environment (S1–5), human feces (H1–5), and surface water (W1–5) (Kovač et al., 2018; Supplementary Table 1). The *B. subtilis* and *C. jejuni* were co-cultivated at 42°C under microaerobic conditions for 24 h in static cultures. The densities (CFU/mL) of *C. jejuni* and *B. subtilis* in the monocultures and co-cultures were determined by plating the cultures on selective media and incubating the plates at the appropriate temperatures under microaerobic and aerobic conditions, respectively. Co-cultivation experiments were carried out as three biological replicates, with up to three technical replicates.

### *Bacillus subtilis* Spore Preparation

*Bacillus subtilis* spores were prepared according to Warriner and Waites (1999), with some modifications. Briefly, an overnight culture of *B. subtilis* prepared in LB media (200 rpm, 37°C) was inoculated (at 1% inoculum) into sporulation medium, which contained 16 g/L Nutrient broth (Oxoid, United Kingdom), 2 g/L KCl (Thermo Fisher Scientific, United States), 1 mM MgSO_4_ (Oxoid, United Kingdom), 1 mM CaCl_2_ (Merck, Germany), 1 μM FeSO_4_ (Sigma Aldrich, Germany), 10 μM MnCl_2_ (Sigma Aldrich, Germany) and 2.8 mM D-(+)-glucose (Sigma Aldrich, Germany), and incubated for 4 days (200 rpm, 37°C). The culture was treated at 80°C for 30 min and washed with 0.9% NaCl (10,000 × *g*, 10 min) three times before being stored in 10% glycerol at –20°C. The spore concentrations (as CFU/mL) were determined before freezing and after thawing, prior to the start of the experiments.

### Determination of *Bacillus subtilis* Susceptibility to Antimicrobials

Minimal inhibitory concentrations (MICs) for eight antimicrobials of human or veterinary importance were determined against two *B. subtilis* strains, *B. subtilis* PS-216 and *B. subtilis* ATCC 6633, by the broth microdilution method using MH broth. Twofold serial dilutions of the antibiotics tetracycline (Fluka, Switzerland), chloramphenicol (Calbiochem, United States), kanamycin (Calbiochem, United States), erythromycin (Sigma, United States), streptomycin (Sigma, United States), gentamycin (Glentham Life Science, United Kingdom), tylosin tartrate (Sigma, United States) and ampicillin (Roche, Germany) were prepared at concentrations ranging from 0.5 to 64 μg/mL. The assays were performed in 96-well microtiter plates. For each well, 50 μL of the corresponding dilutions of the antibiotics were added to 50 μL bacterial suspension previously diluted in MH broth to 10^5^ CFU/mL from an overnight culture (LB broth, 37°C, 16 h, aerobic conditions, 200 rpm). The MICs are expressed as the lowest concentration of antibiotic at which no visible growth of bacteria occurred (i.e., absence of turbidity). Breakpoint values as described by the European Food Safety Agency (EFSA, 2012) were used to interpret the MIC results of various antimicrobials against *Bacillus* spp.

### Acid and Bile Salt Tolerance of *Bacillus subtilis* Vegetative Cells and Spores

Resistance of vegetative cells and spores to simulated gastric conditions and bile salts was determined as described previously (Barbosa et al., 2005; Mingmongkolchai and Panbangred, 2018), with some modifications. Briefly, *B. subtilis* overnight cultures (16 h) in Luria-Bertani (LB) medium (Conda, Spain) containing 1% glucose to prevent sporulation were sub-cultured (at 1% inoculum) into LB medium containing 1% glucose, and grown at 37°C with shaking (110 rpm) for 3 h. Vegetative cells were diluted to an OD_600_ of 0.1 in LB medium acidified to pH 2.5 with 2 M HCl and supplemented with 1 mg/mL pepsin from porcine gastric mucosa (Sigma-Aldrich, Switzerland), or in LB medium supplemented with 0.3% (w/v) bile salts (Oxoid, United Kingdom). For spore assays, the spores were diluted in 5 mL 0.85% NaCl adjusted to pH 2.5 and supplemented with 1 mg/mL pepsin, or isotonic buffer (Oxoid, United Kingdom) supplemented with 0.3% bile salts (Oxoid, United Kingdom). Cultures were incubated at 37°C with agitation (110 rpm), and aliquots were removed after 30, 60, and 90 min for acid tolerance, and after 60 and 180 min for bile salt tolerance. Bacterial cell survival (%) was calculated as follows: N_A_/N_B_ × 100, where N_A_ = log CFU/mL after incubation and N_B_ = log CFU/mL before incubation.

### Broiler Chicken Colonization

One-day-old broiler chicks (*n* = 45) were obtained from a commercial hatchery and were divided randomly into four groups with 11 or 12 chicks per group. The broilers were kept in tubs with soft bedding, and water (regular city water) and feed (Purina non-medicated starter feed with nutritional content presented in Supplementary Table 2) provided *ad libitum*. The animals were housed in a room with BSL-2 practices in place at laboratory animal facilities at Iowa State University with standard temperature and lightning recommended by commercial broilers in the region (Supplementary Table 3).

At the age of 8 d, all of the broilers were inoculated with 4 × 10^6^ CFU *C. jejuni* 11168 by oral gavage. Successful colonization was confirmed by cloacal swabs 5 days after inoculation.

To evaluate the use of *B. subtilis* PS-216 spores to prevent and/or reduce *C. jejuni* colonization, the broilers were administered one of the following *B. subtilis* treatment regimens: (i) No treatment Control (*n* = 12), as inoculated with *C. jejuni*, but not treated with *B. subtilis* PS-216 spores; (ii) Pre-treatment group (*n* = 11), as treated with *B. subtilis* PS-216 spores before inoculation with *C. jejuni*, from an age of 1 to 7 days; (iii) Continuous treatment (*n* = 11), as treated with *B. subtilis* PS-216 spores throughout the experiment, from an age of 1 to 20 days; and (iv) Post-treatment group (*n* = 11), as treated with *B. subtilis* PS-216 spores 5 days after inoculation with *C. jejuni*, from an age of 13–20 days. The *B. subtilis* PS-216 spore treatments were administered in the drinking water at approximately 2.5 × 10^6^ CFU/mL water *B. subtilis* PS-216 spores on the corresponding days as given above. The spore-containing drinking water was given to the broiler chickens *ad libitum.*

Cloacal swabs were collected from each broiler prior to *C. jejuni* inoculation (day 0) to confirm the absence of *C. jejuni*, 5 days after inoculation (day 5) to confirm colonization with *C. jejuni*, and 8 and 11 days after inoculation. At 21 days of age, all of the broilers were sacrificed and weighted, and the cecum contents were collected. To enumerate *C. jejuni* in feces, all of the swabs and cecum contents collected were diluted 10-fold in MH broth, and then plated onto MH-BSS and treated at 80°C for 30 min, and plated onto HiChrom *Bacillus* agar to enumerate the *Bacillus* spores.

### Statistical Analysis

One-way ANOVA with Tukey’s *post-hoc* tests was used to analyze the effects of different *B. subtilis* strains on *C. jejuni* NCTC 11168. To evaluate the effects of co-culture on *B. subtilis* growth, Student’s *t*-tests (paired) were used. Two-way ANOVA with Bonferroni *post-hoc* tests was used to analyze the effects of *B. subtilis* PS-216 on multiple *C. jejuni* strains. The differences between treated and untreated broiler groups were analyzed using Student’s *t*-tests. Statistical analysis was performed with the SPSS software, version 21 (IBM Corp., NY, United States) and GraphPad Prism software, version 8 (GraphPad Software Inc., CA, United States). A *P*-value < 0.05 was considered statistically significant.

## Results

### *Bacillus subtilis* Reduces Growth of *Campylobacter jejuni* in Co-culture

**FIGURE 1:**
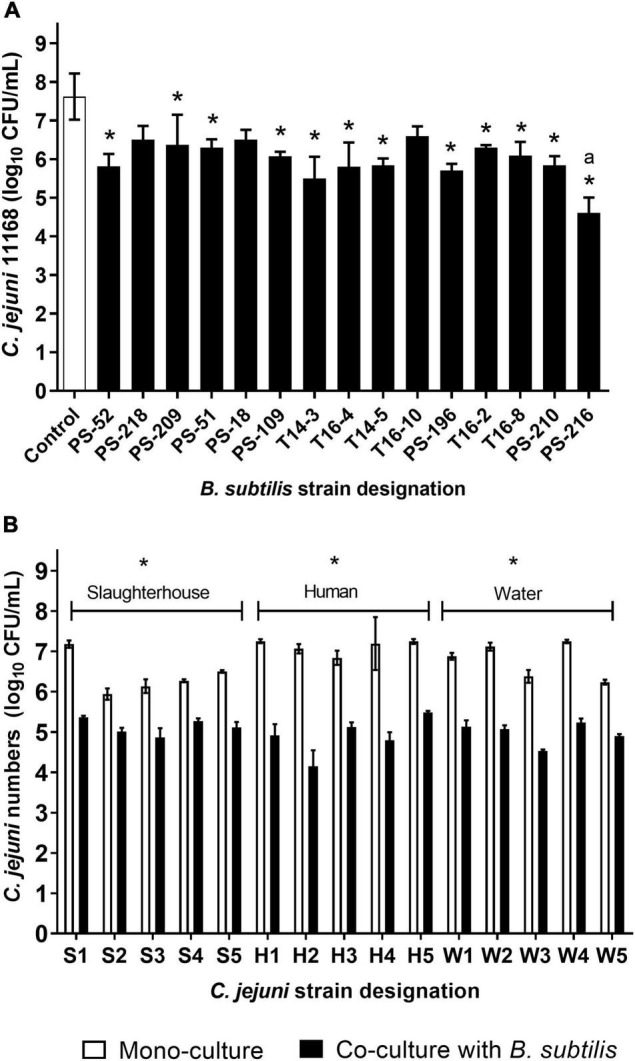
Anti-*Campylobacter* activities of the 15 *B. subtilis* strains against *C. jejuni* NCTC11168 **(A)** and of *B. subtilis* PS-216 against 15 *C. jejuni* strains **(B)**. Data are means ± standard deviation from three replicates. *P < 0.05, vs. relevant control (one-way ANOVA with Tukey’s *post-hoc* tests).

To determine and select the most suitable *B. subtilis* strain against *C. jejuni*, we initially analyzed the anti-*Campylobacter* activities against *C. jejuni* NCTC11168 of 15 *B. subtilis* strains isolated from riverbank soil in Slovenia (Štefanič and Mandic Mulec, 2009) and from tomato rhizosphere (Oslizlo et al., 2015). The majority of the *B. subtilis* strains showed substantial anti-*Campylobacter* effects, with significant reductions in CFU/mL of *C. jejuni* (*P* < 0.05). Only three *B. subtilis* strains had no effects on the growth of *C. jejuni* (*P* > 0.05): PS-218, PS-18, and T16-10. Compared to *C. jejuni* mono-cultures, the co-cultures with *B. subtilis* for 24 h reduced *C. jejuni* by 1.03–3.01 log CFU/mL (Figure 1A). Among the *B. subtilis* strains, PS-216 showed the highest reduction of *C. jejuni* 11168 (3.01 log CFU/mL), and was therefore selected for the further experiments (Figure 1A).

To confirm the efficacy of *B. subtilis* PS-216 against *C. jejuni*, it was further tested by co-culture against 15 *C. jejuni* strains isolated from various sources. Significant reductions in *C. jejuni* by *B. subtilis* PS-216 were seen, which ranged from 0.93 to 2.81 log CFU/mL (*P* < 0.05) across the different *C. jejuni* strains (Figure 1B). *B. subtilis* PS-216 was most effective against *C. jejuni* strains isolated from human feces, with an average reduction of 2.22 ± 0.45 log CFU/mL, and was least effective against strains from the slaughterhouse environment, with an average reduction of 1.28 ± 0.32 log CFU/mL (Figure 1B). No significant differences in the growth of *B. subtilis* were seen when the *B. subtilis* strains were grown in co-cultures with *Campylobacter* or grown in monocultures (Supplementary Figure 1). Although the co-cultivation conditions were favorable for *C. jejuni* according to the higher starting numbers and microaerobic environment, these results showed that *B. subtilis* PS-216 had broad anti-*Campylobacter* activity against these different *C. jejuni* strains of various origins.

### *Bacillus subtilis* PS-216 Is Susceptible to Antimicrobials

For a probiotic to be considered safe for use in human and animal nutrition, it should not harbor any transferable antibiotic resistance genes (FEEDAP, 2012; Pariza et al., 2015). Thus, the antimicrobial susceptibilities of *B. subtilis* PS-216 to eight antimicrobials relevant to human and veterinary medicine were examined. The antibiotics tested were tetracycline, chloramphenicol, kanamycin, erythromycin, streptomycin, gentamycin, tylosin tartrate and ampicillin, and the reference strain *B. subtilis* ATCC 6633 was used as a control in the MIC assays. Both *B. subtilis* PS-216 and the reference strain were susceptible to all of the antimicrobials tested, with the MICs for tetracycline, streptomycin and ampicillin against *B. subtilis* PS-216 > 16-fold, 2-fold, and > 4-fold higher than those in the reference strain (Table 1).

**TABLE 1:** Susceptibility of *B. subtilis* PS-216 and reference strain *B. subtilis* ATCC 6633 to antibiotics.

*B. subtilis* strain	Antibiotic MIC (mg/L) and strain sensitivity (S/R)^a^							
	
	TET	CHL	KN	ERY	STR	GEN	TY	AMP
PS-216	8 (S)	2 (S)	<0.5 (S)	<0.5 (S)	8 (S)	<0.5 (S)	<0.5	2
ATCC 6633	<0.5 (S)	2 (S)	<0.5 (S)	<0.5 (S)	4 (S)	<0.5 (S)	<0.5	<0.5

*^a^S, sensitive; R, resistant; according to European Food Safety Agency (EFSA, 2012) for Bacillus spp. TET, tetracycline; CHL, chloramphenicol; KN, kanamycin; ERY, erythromycin; STR, streptomycin; GEN, gentamycin; TY, tylosin tartrate; AMP, ampicillin.*

### *Bacillus subtilis* PS-216 Spores Are Highly Resistant to Simulated Gastric Conditions

For a probiotic to be effective against *C. jejuni*, it must survive the harsh gastrointestinal conditions (i.e., acid and bile salts) to reach the colonization site of *C. jejuni* in the intestine. The acid and bile salt tolerance assays showed that the vegetative cells of *B. subtilis* PS-216 were very susceptible to simulated gastric conditions, with 100% loss of cell viability when exposed to simulated gastric conditions at 37°C for 30 min (Table 2). In contrast, the spores of *B. subtilis* PS-216 showed 100% survival after 90 min of exposure to simulated gastric conditions and after 180 min in 0.3% bile salts. This showed that the spores of *B. subtilis* PS-216 have excellent resistance to simulated gastric conditions and to 0.3% bile salts, which suggests that the spore form of *B. subtilis* PS-216 can be used as a probiotic in chickens.

**TABLE 2:** Survival of *B. subtilis* PS-216 vegetative cells and spores under simulated gastric conditions (1 mg/mL pepsin, pH 2.5) and with 0.3% bile salts, presented as % of cells/spores after treatment.

Treatment	Time	Survival (%)	
		
Condition	(min)	Vegetative cells	Spores
Gastric	30	0	100
	60	0	100
	90	0	100
Bile salts	60	0	100
	180	0	100

### *Bacillus subtilis* PS-216 Reduces *Campylobacter jejuni* Colonization in Broiler Chickens

**FIGURE 2:**
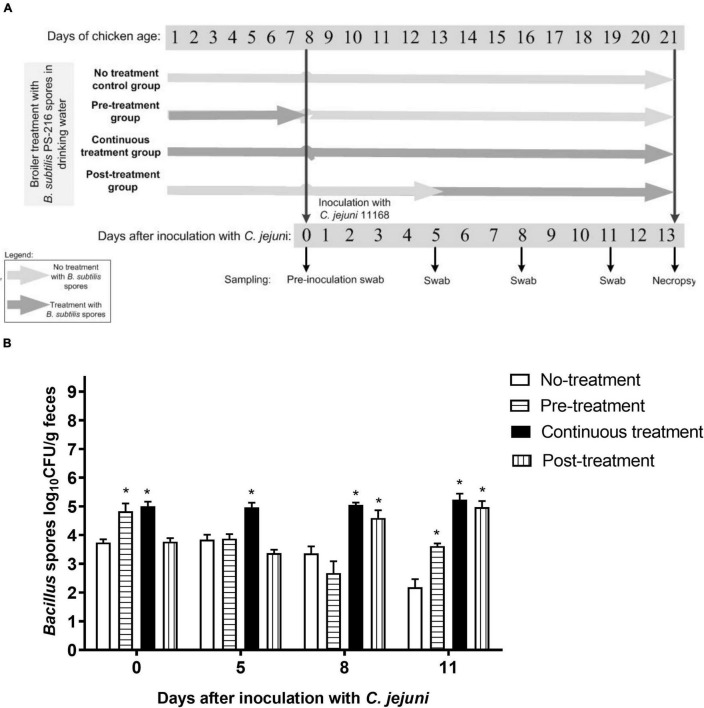
Schematic representation of broiler colonization with *C. jejuni* 11168 and *B. subtilis* PS-216 treatments **(A)**; and detection of *Bacillus* spores in the broiler feces according to the *B. subtilis* PS-216 treatments given. Data are means ± standard deviation. *P < 0.05 vs. untreated controls (Student’s *t*-tests) **(B)**.

To evaluate the effects of *B. subtilis* PS-216 on *C. jejuni* colonization in broiler chickens, *B. subtilis* spore solutions (2.5 × 10^6^ CFU/mL water) were administered to broilers via their drinking water. The broilers underwent the following treatment regimens (Figure 2A): (i) Control group, inoculated with *C. jejuni* but not treated with *B. subtilis* PS-216 spores; (ii) Pre-treatment group, treated with *B. subtilis* PS-216 spores 7 days prior to *C. jejuni* inoculation (*B. subtilis* PS-216 as a preventive measure); (iii) Continuous treatment group, treated with *B. subtilis* PS-216 spores for the duration of the experiment (21 days); and (iv) Post-treatment group, treated with *B. subtilis* PS-216 spores 8 days after the broilers were colonized with *C. jejuni* (*B. subtilis* PS-216 as a curative measure).

All of the broilers had baseline *Bacillus* spore counts in their feces (3.8 × 10^3^ CFU/g feces) at placement when they were 1 day old, and as expected, the *Bacillus* spore counts were higher when the broilers were given *B. subtilis* PS-216 spores in their drinking water (Figure 2B). Furthermore, these higher *Bacillus* spore counts (> 5 × 10^4^ CFU/g feces) dropped abruptly after treatment cessation. This is exemplified by the Pre-treatment group of broilers, where high *Bacillus* spore counts were detected at first sampling during the *B. subtilis* PS-216 spore treatment (day 0 after inoculation with *C. jejuni*), with a drop to baseline (comparable to untreated Control group) after treatment cessation (second sampling, day 5 after inoculation with *C. jejuni*) (Figure 2B). For the last feces sampling day (day 11 after inoculation with *C. jejuni*), all of the treated groups (Pre-treatment, Continuous treatment, Post-treatment) showed higher *Bacillus* spore counts compared to the untreated Control group (by 1.57, 3.19, 2.94 log CFU/g feces, respectively). *Campylobacter* counts showed no significant differences between groups at cloacal swab samplings (Supplementary Figure 2).

**FIGURE 3:**
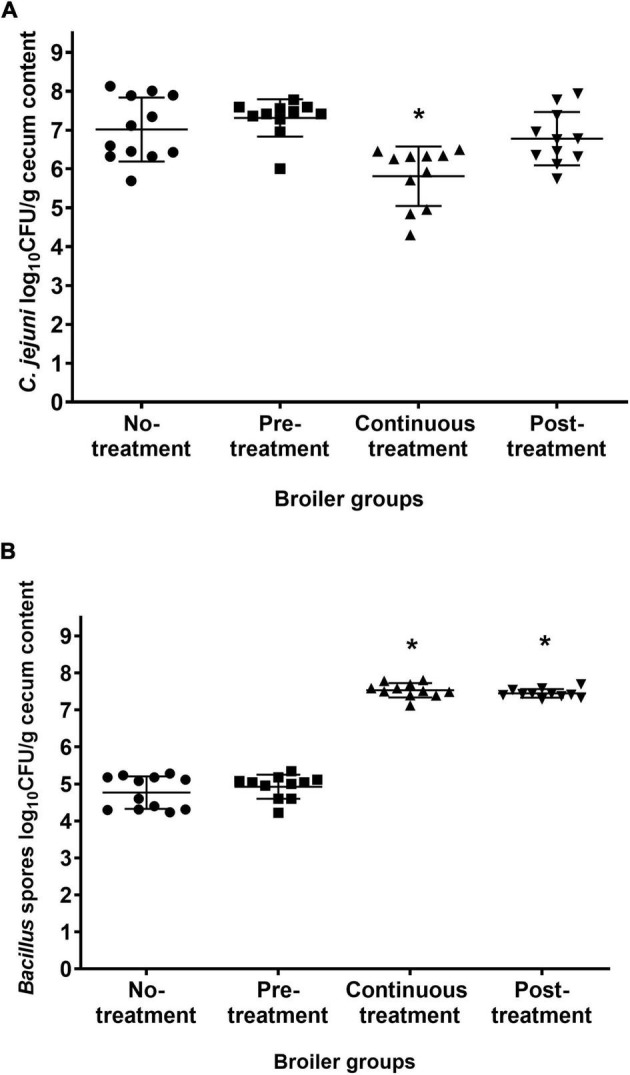
Cecum contents of *C. jejuni* **(A)** and *Bacillus* spores **(B)** of the broilers 21 days of age according to the *B. subtilis* PS-216 treatments given. Each symbol represents an individual broiler chicken. Horizontal line, means ± standard deviations. *P < 0.01 vs. untreated control (Student’s *t*-tests).

At 21 days of age, all of the broilers were sacrificed, and their cecum contents were examined for *C. jejuni* and for *Bacillus* spores. *C. jejuni* was detected in all of the broilers, regardless of the treatment regimens (Figure 3A). However, compared to the untreated Control group, there was a significant decrease in *C. jejuni* counts in the cecum contents in the group continuously treated with *B. subtilis* PS-216 spores (*P* = 0.002), with a mean decrease of 1.2 log CFU/g feces in the *C. jejuni* counts. In contrast, the *C. jejuni* counts in the Pre-treatment and Post-treatment groups were comparable to the untreated Control group. This indicates that the continuous supplementation of broilers with *B. subtilis* PS-216 spores is an effective measure to lower the CFU counts of *C. jejuni* in the caeca.

For the *Bacillus* spore counts, the 7-days pre-treatment of the broilers with *B. subtilis* PS-216 spores had no effects on the final *Bacillus* spore counts in the cecum, as this was comparable to the untreated Control group (Figure 3B). However, the *Bacillus* spore counts in the broilers treated with *B. subtilis* PS-216 spores until the necropsy time (Continuous treatment, Post-treatment) were higher than those in the untreated Control group (increases of 2.76, 2.68 log CFU/g feces, respectively; *P* < 0.05).

### *Bacillus subtilis* PS-216 Spore Treatment Increases Weigh Gain in Broilers

**FIGURE 4:**
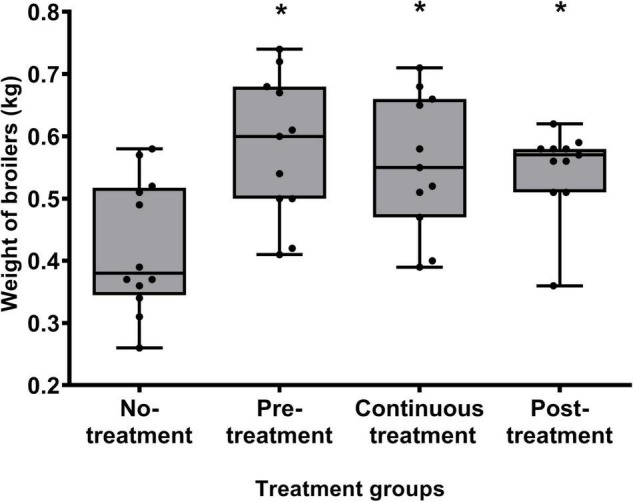
Body weights of the broilers at 21 days of age according to the *B. subtilis* PS-216 treatments given. Each symbol represents an individual broiler chicken. Data are shown as box plots with whiskers. *P < 0.05 vs. untreated control (Student’s *t*-tests).

The body weights of the broilers were measured at the end of the study to determine whether the water supplementation of *B. subtilis* PS-216 spores affected the weight gain of the broilers. Indeed, the weights of the broilers in the three groups treated with *B. subtilis* PS-216 spores were significantly higher than those of the untreated Control group (Figure 4; *P* < 0.05). An average increase of 158 g was measured for the Pre-treatment group, 134 g for the Continuous treatment group, and 124 g for the Post-treatment group, which represented further weight increases over the non-treated Control group of 37, 32, and 30%, respectively.

Altogether, these data showed that treatment of the broilers with *B. subtilis* PS-216 spores in their drinking water significantly reduced the CFU/g of *C. jejuni* in cecum content and increased the bodyweight of these broiler chickens.

## Discussion

The food-borne pathogen *C. jejuni* is among the most prevalent bacterial causes of gastroenteritis in the EU and the United States (Tam and O’Brien, 2016; Kuhn et al., 2020). Although *C. jejuni* can be spread through various routes, the main source of *C. jejuni* is the reservoir in chickens. Thus, control of *C. jejuni* at the farm level should help to reduce the risk of *C. jejuni* spread through the food chain. Probiotics (i.e., live-fed microbials) can provide benefits to the animal host, and if shown to be effective for the reduction of *C. jejuni*, they can be used as a pre-harvest intervention on farms (van Wagenberg et al., 2020). In the present study, we identified *B. subtilis* PS-216 as an additive that can be given to broiler chickens in the form of spores in the drinking water to reduce *C. jejuni* colonization and to increase the broiler weight gain.

Previous studies have reported that probiotic bacteria can show species-specific and strain-specific anti-*Campylobacter* activities both *in vitro* and in the chicken host (Mohan, 2015; Mortada et al., 2020). Moreover, Wine et al. (2009) reported that one *Lactobacillus* strain was effective for the inhibition of *C. jejuni*, while another was not, while the same two strains did not have the same effects on two different *C. jejuni* strains. We have shown a similar pattern here, although not to the same extent, in the anti-*Campylobacter* activity of different *B. subtilis* strains. Of the 15 *B. subtilis* strains tested, the majority (12 of 15) inhibited the growth of *C. jejuni* 11168 to various levels. Nevertheless, *B. subtilis* PS-216 had superior effects compared to all of the other *B. subtilis* stains tested, and thus the activity of *B. subtilis* PS-216 was additionally tested, and confirmed, against 15 different *C. jejuni* strains that originated from three different environments. Contrary to Wine et al. (2009), we observed a similar activity of PS-216 against all of the *C. jejuni* strains tested; i.e., all of the reductions in *C. jejuni* were significant regardless of the *C. jejuni* origin. This confirmed the broad anti-*Campylobacter* effects of *B. subtilis* PS-216 *in vitro*, and highlights the potential of this strain for use in high-load *Campylobacter* environments, such as the poultry intestinal tract.

However, before a probiotic can be used commercially, it must first meet additional standards. One of these requirements is susceptibility to antibiotics, as probiotics can carry antibiotic resistance determinants that can be transferred to other residential bacteria in the gut via horizontal gene transfer, which will amplify the growing problem of antibiotic resistance (Sharma et al., 2014; Zheng et al., 2017; EFSA and ECDC, 2019). Thus, it is of utmost importance that probiotics do not serve as genetic reservoirs for the emergence of antibiotic resistance, and consequently do not pose a risk to animal and human health. We tested the *B. subtilis* PS-216 susceptibility to antibiotics according to EFSA guidance (FEEDAP, 2012). *B. subtilis* PS-216 was indeed susceptible to the several antibiotics examined in this study, and therefore complies with the required probiotic standards.

Additionally, to be effective, probiotic strains must reach the gut, and thus they must withstand the harsh conditions of the gastrointestinal tract of an animal. Indeed, the spores of *B. subtilis* PS-216 were highly resistant to simulated conditions of the gastrointestinal tract in this study, and thus should pass through the gastrointestinal tract and into the intestine, where their action against pathogens needs to take place. On the contrary, the vegetative cells of *B. subtilis* PS-216 did not survive these gastric conditions, and are thus not suitable for such probiotic use. Cartman et al. (2008) showed that *B. subtilis* spores germinate and proliferate in the chicken intestine. Our results also suggest that *B. subtilis* PS-216 germinates and proliferates in the broiler intestine, as the *Bacillus* CFU/g counts of cecum samples at day 21 were approximately 1 log higher than in the inoculation solution. Thus, only the spores need to survive gastric conditions to have a probiotic effect.

A further requirement that a potential probiotic must meet is *in vivo* efficacy, as the efficacy of a probiotic cannot be judged by *in vitro* results alone (Robyn et al., 2013; Mortada et al., 2020). Here, both the *in vitro* and *in vivo* results showed a significant reduction in *Campylobacter* for the treatment with *B. subtilis* PS-216 spores. The *B. subtilis* PS-216 spores were added to broiler chicken drinking water as a treatment solution: (i) Before inoculation with *C. jejuni*, to test this as a preventive measure; (ii) after established colonization with *C. jejuni*, to test this as a therapeutic measure; and (iii) continuously from day 1. This animal study yielded three important conclusions: (i) The *Bacillus* spore counts in the feces increased during the *B. subtilis* PS-216 spore treatment, but decreased after the treatment ended; (ii) continuous *B. subtilis* PS-216 spore treatment reduced *C. jejuni* in the cecum of broilers, although the preventive (Pre-treatment) and therapeutic (Post-treatment) uses of *B. subtilis* were not effective at the *B. subtilis* PS-216 spore concentration used here (10^6^ CFU/mL water); and (iii) short-term treatment at the beginning or end of the experiment increased the broiler weights compared to the untreated controls. Previously, Latorre et al. (2014) reported that only continuous treatments of chickens with *B. subtilis* spores led to high spore counts in the chicken intestine. This is in line with our results, where there was a decrease in the *Bacillus* spore counts in feces and cecum after short-term *B. subtilis* PS-216 spore treatment, but a constant *Bacillus* spore count when the *B. subtilis* PS-216 spores were added continuously. An increase in *Bacillus* spore counts after the addition of *B. subtilis* spores was also shown by Ciurescu et al. (2020); however, the level of *Bacillus* spore counts in the treated groups in the cecum content were different from the present study. We determined *Bacillus* spore counts of around 10^7^ CFU/g feces, while Latorre et al. (2014) observed lower *Bacillus* spore counts, of approximately 10^5^ CFU/g feces. Interestingly, the number of *Bacillus* spores in the cecum was also higher than in the feces, and higher than the concentration of *B. subtilis* PS-216 spores added to the drinking water (i.e., the treatment solution). This suggests that the *B. subtilis* PS-216 spores either germinate in the intestine into vegetative cells that then die off before being passed as feces, or the *B. subtilis* PS-216 spores somehow accumulate in the broiler cecum. Nevertheless, the high spore counts without continuous administration were not sufficient to reduce the number of *C. jejuni* in the cecum of the treated broilers. Continuous treatment with *B. subtilis* PS-216 spores (10^6^ CFU/mL water) resulted in a significant reduction in *C. jejuni* (1.2 log CFU/g feces) that was comparable to multispecies probiotics containing *B. subtilis* tested by Arsi et al. (2015) (up to 3 log CFU/g feces reduction), multi-strain *B. subtilis* probiotics tested by Aguiar et al. (2013) (from 1 to 4 log CFU/g feces reduction), and a single species *B. subtilis* probiotic Calsporin (Guyard-Nicodème et al., 2016) (1.7 log CFU/g reduction). Differences in *B. subtilis* efficacy in broilers can be attributed to strain variability, as well as to treatment variability.

One possible mechanism of anti-*Campylobacter* action of *B. subtilis* is competitive exclusion, in which the probiotic occupies the *C. jejuni* attachment sites in the intestine, thus preventing *C. jejuni* adhesion (Wine et al., 2009). *B. subtilis* strains have been shown before to reduce pathogens like *E. coli* (La Ragione et al., 2001) and *Salmonella* (La Ragione and Woodward, 2003) in the intestine by means of competitive exclusion. Some *B. subtilis* strains have been shown to promote the colonization of *Lactobacillus* in chicken intestine (Park et al., 2017), thereby acidifying the intestine and making it an unfriendly environment for *C. jejuni*. *B. subtilis* is also known to produce antimicrobials (Tamehiro et al., 2002; Huang et al., 2009; Efremenkova et al., 2016) that might act in the gut, although it is not clear whether these would be expressed in the broiler intestine. In previous studies, we showed that *B. subtilis* PS-216 can act against the reference *C. jejuni* NCTC 11168 strain in variable settings by producing the antimicrobial substance bacillaene (Erega et al., 2021; Šimunović et al., 2022); however, we have no data at this stage to support the hypothesis that this antimicrobial affects the survival of *C. jejuni* in these broiler chickens.

Adding probiotics as feed/water supplements represents a good *Campylobacter* control strategy (Koutsoumanis et al., 2020). *B. subtilis* probiotics have been shown to have immunomodulatory effects in the gut and to modulate the gut microbiota (Hayashi et al., 2018), promote muscle development and meat quality (Zhou et al., 2015), and promote growth, feed conversion and body weight in chickens with potential anti-pathogenic effects, due to their good enzymatic activity (Fritts et al., 2000; Hmani et al., 2017; Park et al., 2017). Although such an intervention might increase production costs somewhat, and consequently the cost of the food on the market, it can also be a cost-effective intervention when the addition of probiotics reduces the risk of *Campylobacter* and simultaneously increases the growth performance (van Wagenberg et al., 2020). All of the *B. subtilis* PS-216 spore treatments in the present study resulted in increased body weight of these broiler chickens. Remarkably, even the 8-days treatment resulted in a significant weight improvement. Thus, this use of *B. subtilis* PS-216 spores offers the poultry industry different options: short-term use that can be implemented to increase the weight of broilers, and continuous use that on top of the weight increase, can also reduce *C. jejuni* counts and improve the safety of the food. In conclusion, our study suggests that *B. subtilis* PS-216 spores represent a new and attractive solution to enhance broiler chicken health and to increase poultry and food safety.

To conclude, these results underline the potential of *B. subtilis* PS-216 spores as a treatment for improving poultry production and food safety. The anti-*Campylobacter* activity of *B. subtilis* PS-216 was demonstrated by *in vitro* and *in vivo* experiments in this study. In addition to its anti-*Campylobacter* activity, *B. subtilis* PS-216 spore treatments also increased the weight gain of the broiler chickens. Although *B. subtilis* might also act against other pathogenic bacteria, this was not tested in the present study, and future studies are needed to confirm the overall beneficial effects of *B. subtilis* PS-216 spores for enhanced poultry health and performance. In the future, it will also be necessary to evaluate the anti-*Campylobacter* effects *of B. subtilis* PS-216 under different settings, to determine its reproducibility and broader applicability as a viable intervention measure for *Campylobacte*r in primary poultry production.

## Data Availability Statement

The original contributions presented in this study are included in the article/Supplementary Material, further inquiries can be directed to the corresponding author.

## Ethics Statement

All of the animal protocols and procedures used in this study were reviewed and approved by the Institutional Animal Care and Use Committee at Iowa State University before the start of the experiments (IACUC Protocol IACUC-18-322).

## Author Contributions

KŠ, PŠ, AK, QZ, OS, IMM, and SSM: conceptualization. KŠ, PŠ, and AK: methodology and validation. KŠ: formal analysis, data curation, writing—original draft preparation, and visualization. KŠ, AE, and OS: investigation. IMM, SSM, and QZ: resources. PŠ, AK, IMM, SSM, QZ, and OS: writing—review and editing and supervision. IMM, SSM and AK: project administration and funding acquisition. All authors have read and agreed to the published version of the manuscript.

## Conflict of Interest

The authors declare that the research was conducted in the absence of any commercial or financial relationships that could be construed as a potential conflict of interest.

## Publisher’s Note

All claims expressed in this article are solely those of the authors and do not necessarily represent those of their affiliated organizations, or those of the publisher, the editors and the reviewers. Any product that may be evaluated in this article, or claim that may be made by its manufacturer, is not guaranteed or endorsed by the publisher.
