# Supplementary material for: Bacillus subtilis PS-216 Spores Supplemented in Broiler Chicken Drinking Water Reduce Campylobacter jejuni Colonization and Increases Weight Gain
Source: Front Microbiol. 2022 Jul 8;13:910616. doi: 10.3389/fmicb.2022.910616 (PMC9304915; doi:10.3389/fmicb.2022.910616)
Supplement: Supplementary file 1 [file Table_1.pdf]

Supplementary Material

1 Supplementary Figures

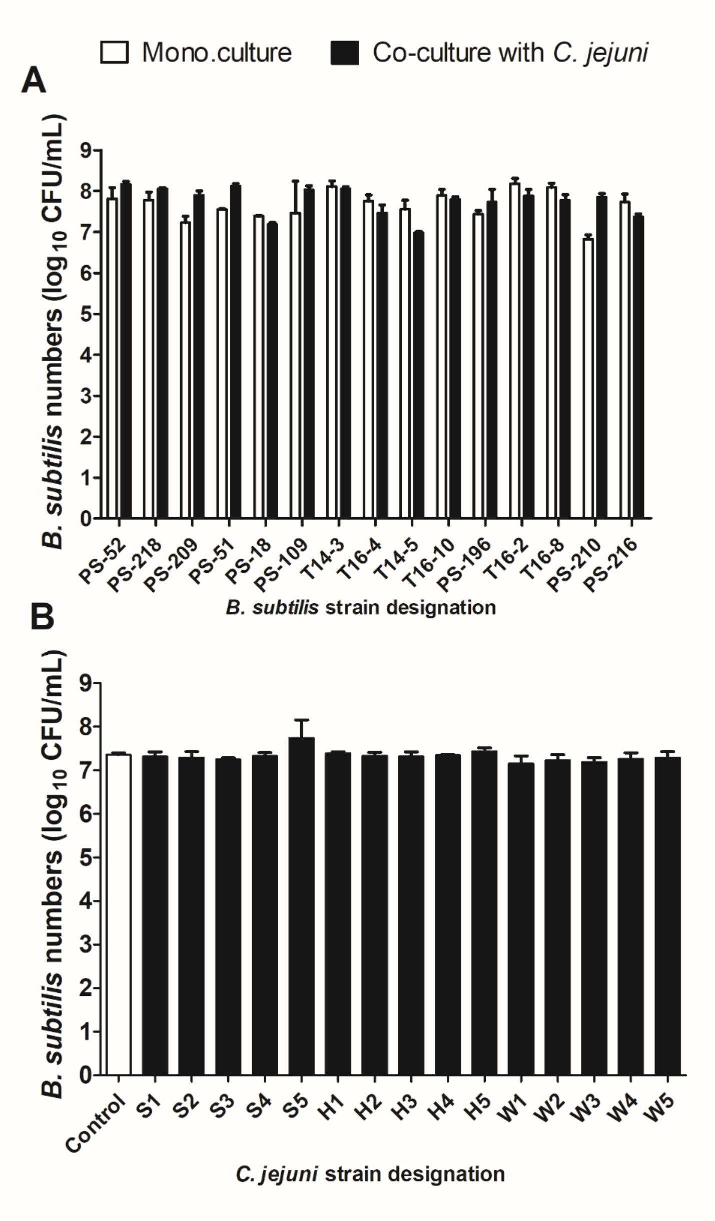

Supplementary Figure S1. Effects of co-cultivation of *C. jejuni* 11168 and *B. subtilis* on growth of the 15 *B. subtilis* strains (**A**) and co-cultivation of 15 *C. jejuni* strains on growth of *B. subtilis* PS-216 (**B**). Data are means ±standard deviation from three replicas. No significant differences were seen versus the relevant controls (one-way ANOVA with Tukey's post-hoc tests).

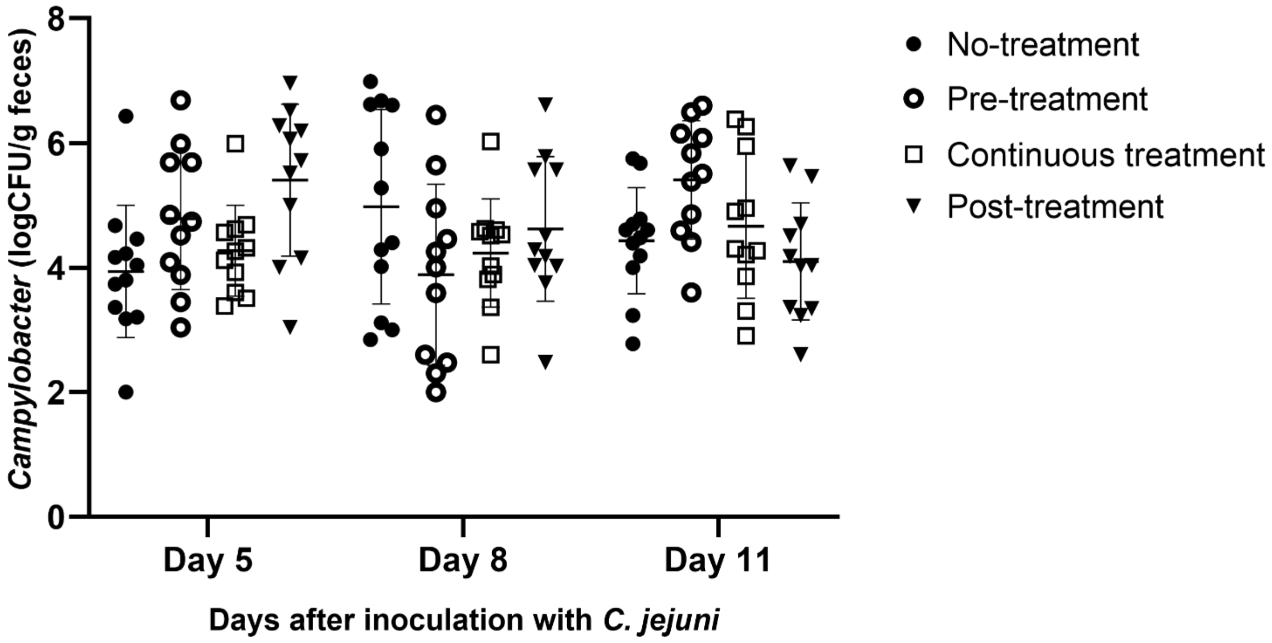

Supplementary Figure S2. Detection of *Campylobacter* counts in the broiler faeces collected with cloacal swabbing, according to the *B. subtilis* PS-216 treatments given. Data are means ±standard deviation. No significant differences were seen versus the relevant controls (one-way ANOVA with Tukey's post-hoc tests).

Supplementary Tables

Table S1. Species, strain designation, sources and references for strains used in the study.

Species	Strain designation	Source	Reference
C. jejuni	11168	NCTC	Kovač et al. (2015)
	H1	Human faeces	Kovač et al. (2018)
	H2		
	H3		
	H4		
	H5		
	S1	Slaughterhouse	Kovač et al. (2018)
	S2		
	S3		
	S4		
	S5		
	W1	Surface water	Kovač et al. (2018)
	W2		
	W3		
	W4		
	W5		
B. subtilis	PS-218	Riverbank soil	Štefanič and Mandič-Mulec (2009)
	PS-209		
	PS-51		
	PS-18		
	PS-109		
	T14-3	Tomato rhizosphere	Oslizlo et al. (2015)
	T16-4		
	T14-5		
	T16-10		
	T16-2		
	T16-8		
	PS-210	Riverbank soil	Štefanič and Mandič-Mulec (2009)
	PS-52		
	PS-216		
	PS-196		

Table S2. Animal feed composition.

Nutrient	Min / Max	Amount
Crude Protein	MIN	18.00 %
Lysine	MIN	0.90 %
Methionine	MIN	0.34 %
Crude Fat	MIN	3.00 %
Crude Fibre	MAX	5.00 %
Calcium (Ca)	MIN	0.75 %
Calcium (Ca)	MAX	1.25 %
Phosphorus (P)	MIN	0.55 %
Salt (NaCl)	MIN	0.25 %
Salt (NaCl)	MAX	0.75 %
Vitamin A	MIN	5000 IU/LB
Vitamin E	MIN	14 IU/LB

Table S3. Animal lighting and temperature (°C) schedule during the study.

Chick Age	Lighting Interval*	Temperature Under Heater in °C
**1**	12:30 AM – 11:30 PM (23 h light)	35 (95F)
**2**	12:30 AM – 11:30 PM	35
**3**	12:30 AM – 11:30 PM	35
**4**	12:30 AM – 11:30 PM	35
**5**	1:30 AM – 10:30 PM (21 h light)	32 (90 F)
**6**	1:30 AM – 10:30 PM	32
**7**	1:30 AM – 10:30 PM	32
**8**	1:30 AM – 10:30 PM	32
**9**	2:30 AM – 9:30 PM (19 h light)	30 (86 F)
**10**	2:30 AM – 9:30 PM	30
**11**	2:30 AM – 9:30 PM	30
**12**	2:30 AM –9:30 PM	30
**13**	2:30 AM – 9:30 PM	30
**14**	2:30 AM – 9:30 PM	30
**15**	2:30 AM – 9:30 PM	30
**16**	3:30 AM – 8:30 PM (17 h light)	28 (82 F)
**17**	3:30 AM – 8:30 PM	28
**18**	3:30 AM – 8:30 PM	28
**19**	3:30 AM – 8:30 PM	28
**20**	3:30 AM – 8:30 PM	28
**21**	3:30 AM – 8:30 PM	28
